# Concrete Performance Produced Using Recycled Construction and By-Product Industrial Waste Coarse Aggregates

**DOI:** 10.3390/ma15248985

**Published:** 2022-12-15

**Authors:** Ali S. Alqarni, Abdulrahman Albidah, Husain Abbas, Tarek Almusallam, Yousef Al-Salloum

**Affiliations:** Chair of Research and Studies in Strengthening and Rehabilitation of Structures, Department of Civil Engineering, King Saud University, Riyadh 11421, Saudi Arabia

**Keywords:** limestone aggregate, quartzite, normal weight aggregate, lightweight aggregate, heavyweight aggregate, recycled aggregate

## Abstract

Concrete is classified as a multi-composite material comprising three phases: coarse aggregate, mortar, and interfacial transition zone (ITZ). Fine and coarse aggregates occupy approximately 70–85% by volume, of which coarse aggregate typically constitutes more than two-thirds of the total quantity of aggregate by volume. The current study investigates the concrete performance produced using various recycled construction and by-product industrial waste coarse aggregates. Six types of coarse aggregates: manufactured limestone, quartzite, natural scoria, by-product industrial waste aggregate, and two sources of recycled concrete aggregates with densities ranging from 860 to 2300 kg/m^3^ and with different strength properties were studied. To determine the coarse aggregate contribution to the overall concrete performance, lean and rich concrete mixtures (Mix 1 and Mix 2) were used. Mix 1 (lean mixture) consisted of a ratio of water to cement (w/c) of 0.5 and cement content of 300 kg/m^3^, whereas a higher quantity of cement of 500 kg/m^3^ and a lower w/c ratio of 0.3 were used for Mix 2 (rich mixture). The results showed that while the compressive strength for different aggregate types in Mix 1 was comparable, the contribution of aggregate to concrete performance was very significant for Mix 2. Heavyweight aggregate produced the highest strength, while the lightweight and recycled aggregates resulted in lower mechanical properties compared to normal weight aggregates. The modulus of elasticity was also substantially affected by the coarse aggregate characteristics and even for Mix 1. The ACI 363R-92 and CSA A23.3-04 appeared to have the best model for predicting the modulus of elasticity, followed by the ACI-318-19 (density-based formula) and AS-3600-09. The density of coarse aggregate, and hence concrete, greatly influenced the mechanical properties of concrete. The water absorption percentage for the concrete produced from various types of aggregates was found to be higher for the aggregates of higher absorption capacity.

## 1. Introduction

Concrete is considered the highest utilized and consumed construction material worldwide. Concrete is a heterogonous and multiphase composite material comprised of coarse aggregate, mortar, and interfacial transition zone (ITZ). Fine and coarse aggregates occupy approximately 70–85% of the total quantity of concrete mixtures by volume [1]. The worldwide consumption of concrete is projected to be around 25 billion tons every year [2], of which coarse aggregate typically constitutes more than 45% of the entire volume in concrete mixtures. The mechanical properties and durability are primarily reliant on the characteristics of the phases as well as the interaction between the components. Meanwhile coarse aggregate properties, including surface texture, shape, and mechanical properties, appear to play a vital role in bonding strength, flowability, and hardened properties [3].

The failure mechanics of normal weight aggregate concrete (NWAC) is dependent on the three phases of concrete: coarse aggregate, cementitious matrix, and ITZ. In normal strength concrete (NSC), which is recognized to have a higher ratio of water to cement (w/c), less cement contents, and assuming rigid coarse aggregate, the failure is initiated at the weakest link of ITZ in the form of micro-cracking, causing a separation between the matrix and coarse aggregate particles and leading to crack propagation around the aggregate particles [4,5,6]. Nevertheless, high-strength concrete (HSC) behaves rather differently, and the coarse aggregate mechanical properties are considered the primary controlling strength factor [3,4,7,8,9]. Stress redistribution takes place between the matrix and coarse aggregate when loaded in compression. Since the mortar–aggregate bond in HSC is much stronger than the aggregate, the failure plane and the fracture occur through the aggregate [3,10]. The mechanical performance of HSC produced with coarse gravel aggregate is typically of inferior quality than that of crushed limestone concrete due to the roughened surface texture and angularity of the limestone aggregate [3]. 

The modulus of elasticity of HSC is also greatly influenced by the coarse aggregate quality, and the literature indicates that a stronger aggregate tends to produce stiffer concrete [6,7,9]. This enhancement in the modulus of elasticity was attributed to an improvement in compressive strength, which in turn resulted in enhancement in the matrix and matrix–aggregate bond or ITZ phase. The effect of coarse aggregate in HSC is more pronounced in the elastic modulus than the compressive strength.

Over the last few decades, lightweight aggregate concrete (LWAC) structural components have attracted the attention of the concrete industry owing to the reduced self-weight of structural members, lower building cost, ease of construction, better thermal properties, and fire and acoustic resistance [11,12,13]. Typical examples of lightweight aggregates (LWAs) in the literature for structural LWAC include expanded clay, shale or pumice, and volcanic tuffs [14]. Several factors have been stated in the literature to play a significant role in the performance of LWAC, including microstructure, gradation, density, thermal conductivity, physical, chemical, and mineralogical compositions, shape, and surface texture. LWAC is classified as a three-phase composite material of coarse aggregate, mortar, and ITZ [15]. The quality of ITZ and elastic compatibility in LWAs mainly dictates the behavior of LWA concrete. The mechanical characteristics of LWAC are influenced by the shape of the LWA. The brittleness and fracture energy were more pronounced in LWAC than in normal aggregate concrete of the same strength [11]. 

Heavyweight aggregate concrete (HWAC) has been widely employed in nuclear power plants, military impact-resistant facilities, medical laboratories, shielding applications, and radioactive facilities due to its ability to provide a protection material and its ability to absorb neutron energy [16,17]. While there is no general consensus on the definition of HWAC, concrete having a density varying from 2600 kg/m^3^ to 3840 kg/m^3^ has been classified in the literature as heavyweight concrete [18]. Although the HWA density and mechanical characteristics influence the HWAC performance significantly, its physical and chemical properties have a more noticeable effect on the performance of HWAC compared to the NWAC [18]. De Domenico et al. [19] reported that the use of heavyweight electric arc furnace (EAF) slag, as opposed to natural aggregates in reinforced concrete beams, resulted in not only improved flexural strength, shear strength, and ductility but also a reduction in the crack width, which was attributed to the enhanced characteristics of EAF concrete. Ahmad et al. [20] reviewed the use of ground granulated blast furnace slag (GGBS) as a replacement of cement in improving the properties of concrete.

Risks of natural aggregate resource depletion and moving towards sustainable and environmentally green concrete have promoted the utilization of industrial waste and recycled concrete aggregate (RCA). However, while the concrete performance at the fresh and hardened states appears to be negatively influenced at the full replacement level, its adequacy and compliance with international concrete specifications are met when used at an appropriate replacement level or when treatments are applied. The characteristics of adhered mortar, which is composed of unhydrated and hydrated cement paste, play a major role in the concrete performance at the fresh and hardened states. The adhered mortar typically results in inferior concrete performance, higher porosity, higher water absorption, lower density, lower specific gravity, higher fine content, and lower resistance to abrasion and impact [21,22]. Concrete performance at the fresh and hardened states produced using RCA has been extensively studied [23]. For example, the introduction of additional water into the concrete mixture by roughly 10% was needed in a study [24] for the RCA to achieve similar workability to the concrete produced using natural aggregate. Although there is generally a proportional relationship between the replacement levels of RCA and the degradation in concrete performance, several factors such as porosity, adhered matrix–aggregate bond, ITZ or matrix–aggregate interface, crushing process, and virgin aggregate characteristics play a substantial role in the fresh, mechanical, and durability performance of concrete. Moreover, the quality of concrete being demolished and recycled appears to influence the quality of concrete produced with that RCA. 

It is evident that the effect of coarse aggregate characteristics on concrete performance has been previously investigated and shown to have an enormous impact on concrete performance. Nevertheless, a great deal of these studies considered aggregates of the same density category or looked at the effect of replacement levels with normal aggregates. Limited studies have investigated the performance of concrete produced using various sources of recycled construction and by-product industrial waste coarse aggregates at a full replacement level, and compared the performance with natural or manufactured lightweight and normal weight coarse aggregates. Additionally, there are limited studies on the stress–strain relationships of concrete produced using recycled construction and by-product industrial waste coarse aggregates, especially with a wide range of densities. The current study investigates concrete performance in terms of compressive strength, splitting tensile strength, stress–strain characteristics, and water absorption produced using various recycled construction and by-product industrial waste coarse aggregates. The novelty of the study is covering a wide variety of commonly used coarse aggregates: manufactured limestone, quartzite, volcanic scoria, by-product industrial waste aggregate, and two sources of RCAs with densities ranging from 860 to 2300 kg/m^3^ and with different strength properties were studied. The applicability of the available equations for predicting the elastic modulus of concrete produced using these aggregates is also verified. 

## 2. Experimental Program

### 2.1. Material Properties

Type I Portland cement of Yamama cement factory, Riyadh, Saudi Arabia, conforming to the ASTM C150 requirements [25] with a specific gravity and consistency of 3.15 and 24%, respectively, was utilized in this study. As for the fine aggregate, two sources of fine siliceous sand and crushed limestone coarse sand with a maximum particle size of 4.75 mm were blended at 30% and 70% by mass, respectively, to comply with the ASTM C33 gradation requirements [26].

**Figure 1:**
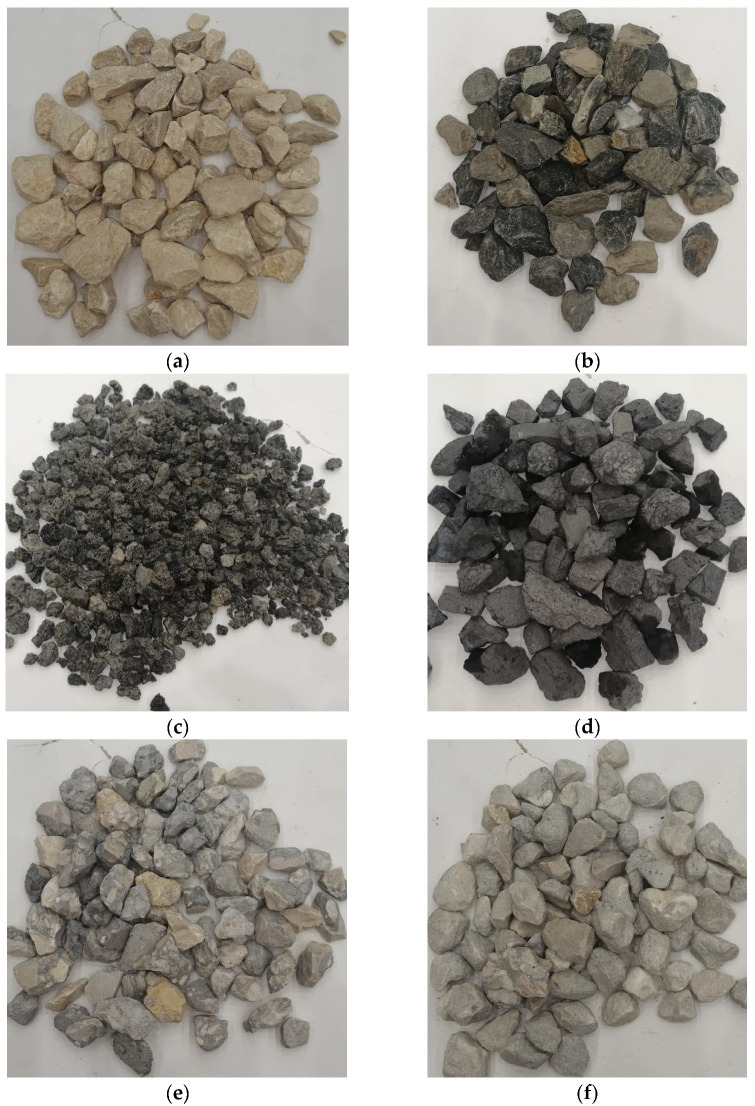
Illustrative pictures of various coarse aggregate types investigated in this study: (**a**) limestone-based normal weight aggregate (LS), (**b**) natural quartzite-based normal weight aggregate (MK), (**c**) volcanic scoria-based LWA, (**d**) by-product industrial waste steel slag-based HWA, (**e**) good quality recycled concrete-based normal weight aggregate (RCA-1), and (**f**) poor quality recycled concrete-based normal weight aggregate (RCA-2).

This study used six types of coarse aggregates covering a broad range of mineralogy and densities, as shown in Figure 1. These coarse aggregates included manufactured limestone-based normal weight aggregate, referred to hereafter as LS, natural quartzite-based normal weight aggregate, referred to as MK, volcanic scoria-based LWA, referred to as LWA, by-product industrial waste steel slag-based heavyweight aggregate (HWA), referred to as HWA, good quality recycled concrete-based normal weight aggregate, referred to as RCA-1, and poor-quality recycled concrete-based normal weight aggregate, referred to as RCA-2. The LS aggregate was obtained from Riyadh, Saudi Arabia. It was manufactured by crushing large rocks into different size fractions, which contained sedimentary rock comprising of chalky crushed limestone, whereas the MK aggregate was located in Makkah, Saudi Arabia, naturally available and characterized as a combination of plagioclase, hard quartzite, and chlorite minerals. The LWA aggregate was procured from Madinah, Saudi Arabia, described as black soft volcanic tuffs of scoria, consisting of pyroxene and sporadic crystals of plagioclase. The HWA aggregate was a by-product resulting from the steel production industrial plants. 

The RCA-1 was produced from low porosity and HSC specimens with compressive strength of roughly 60 MPa collected from the concrete plants. On the other hand, the RCA-2 was prepared from low-strength, highly porous concrete specimens whose compressive strength did not exceed 20 MPa. It should be pointed out that the virgin coarse aggregate source utilized in the concrete specimens of the RCA-1 and RCA-2 was manufactured limestone aggregate (LS), and the primary difference between the two RCAs lies in the quality of adhered mortar in terms of porosity, water absorption, surface texture, and abrasion resistance. The maximum nominal size of coarse aggregate was 20 mm for all aggregate types except for the LWA, with a maximum nominal size of 10 mm. The gradation curves for the various coarse aggregate types satisfied the ASTM C33 requirements [26]. 

The physical properties of aggregates, determined in accordance with the ASTM C127 [27], are summarized in Table 1. The coarse aggregate density utilized in this study varied from 860 kg/m^3^ for the LWA to 2300 kg/m^3^ for the HWA. These aggregate densities are translated into concrete densities ranging from 2025 to 2918 kg/m^3^. The LWA and RCA-2 had very high-water absorptions of 10.6% and 8.2%, respectively, whereas the other aggregates appeared to be in the range of 1.3% to 4.7%.

### 2.2. Concrete Mixture Proportions 

The concrete mixture design considered lean and rich concrete mixtures, referred to throughout the paper as Mix 1 and Mix 2, respectively, to determine the coarse aggregate contribution to the overall concrete performance created using various coarse aggregate types. Mix 1 (lean mixture) consisted of a 0.5 w/c ratio and cement quantity of 300 kg/m^3^, whereas a higher cement quantity of 500 kg/m^3^ and a lower ratio of w/c of 0.3 was used for Mix 2 (rich mixture). The concrete mixture proportions for both mixtures were designed in accordance with ACI 211.1 [28], as shown in Table 2 and Table 3. 

Since the current study is intended to evaluate the effect of various coarse aggregate types on the performance of concrete, several precautions were taken. First, the coarse aggregate-to-fine aggregate ratio by volume was kept fixed at 1.48 for the two concrete mixtures. Second, the relative coarse aggregate volume was maintained fixed at 45% for Mix 1, whereas a lower percentage of 41.3% was used for Mix 2. The minor reduction in the coarse aggregate volume of Mix 2 was due to having a slightly higher quantity of cement compared to Mix 1. These modifications in the concrete mixture design resulted in concrete in which the total aggregate volume occupied was 75.5% for Mix 1 and 69.1% for Mix 2. The concrete mixtures considered the extra water needed for each type of coarse aggregate since they had a wide range of water absorption. In order to address the workability issues related to the mixtures of higher cement quantity and a lower ratio of w/c, especially with the aggregates of higher water absorption, it was essential to utilize MasterGlenium^®^ 51, a water-reducing admixture in compliance with the ASTM C494 Type F [29], which is a BASF product of Germany and was procured from MasterBuilderSolutions, Riyadh, Saudi Arabia. The unit weights of different concrete mixes reported in Table 2 and Table 3 are the experimental values.

The concrete mixtures were all designed to have a slump within a workable range of 75 to 100 mm, which is consistent with the recommended slumps for various types of construction adopted by ACI 211.1 [28]. Therefore, the minimum dosage of superplasticizer was initially added, and the slump test, in accordance with ASTM C143 [30], was performed. In case the slump was not in the desired range, additional superplasticizer dosages were added. It should be pointed out that the superplasticizer dosages were in the range of 1386 to 4159 mL/m^3^ for the lean mixture, and 2311 to 6932 mL/m^3^ for the rich mixture

### 2.3. Specimen Preparation and Testing 

Concrete was poured into 100 × 200 mm^2^ cylinders with proper vibration to reduce air voids. Following the casting process, concrete specimens were removed from the molds one day after the casting day. They were cured for 28 days under a laboratory-controlled environment (24 ± 2 °C) by submerging in curing water tanks until the testing day.

The coarse aggregates (Figure 1) were tested as per the relevant ASTM standards to find out their physical properties, such as water absorption [31], and abrasion resistance by Los Angeles testing [32]. In addition, compressive strength [33], splitting tensile strength [34], and modulus of elasticity tests [35] were carried out in accordance with the ASTM standards to determine the performance of hardened concrete. The water absorption of concrete [36] was used as an indirect measure of the durability of concrete to determine the amount of water being absorbed and penetrated through the depth of concrete.

## 3. Test Results and Discussion

### 3.1. Aggregate Characteristics

The quality of coarse aggregates with respect to the abrasion resistance was assessed using the Los Angeles testing described in the ASTM C131 [32], as illustrated in Table 1. The abrasion results for different aggregates ranged between 13.3% and 45%. The scoria aggregate (LWA) had the least abrasion resistance, with a mass loss of 45%, while the steel slag (HWA) had the highest abrasion resistance, with a mass loss of 13.3%. The recycled aggregates showed different performances owing to the adhered mortar characteristics. Overall, the RCA, whose adhered mortar is characterized to have very porous material, and a high tendency to water absorption, is likely to have less resistance to abrasion and hence poor concrete performance. The mass losses for RCA-1 and RCA-2 were 28.4% and 37.5%, respectively. The LS and MK aggregates had 23.1% and 19.8% mass loss, respectively. 

### 3.2. Compressive Strength 

**Figure 2:**
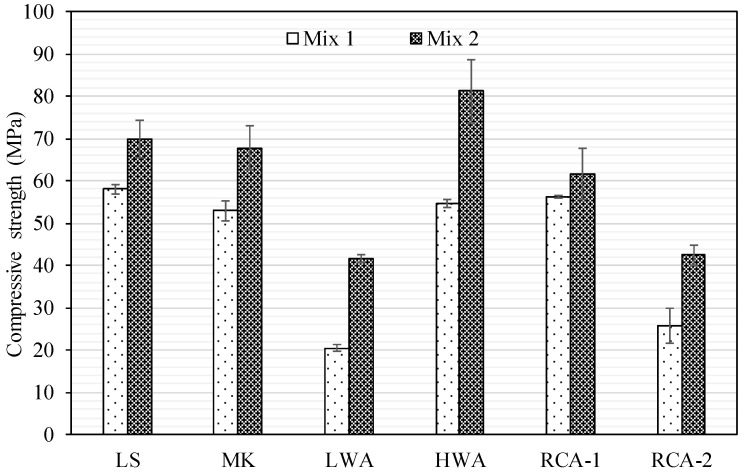
Compressive strength results for Mix 1 and Mix 2.

The compressive strength determined at 28 days for Mix 1 and Mix 2 was performed in accordance with the ASTM C39/C39M-20 [33], and the compressive strength results are illustrated in Figure 2. Generally, lean mix (Mix 1), having a higher ratio of w/c and lower quantity of cement, had lower compressive strength irrespective of the type of coarse aggregate. The concrete compressive strength of Mix 1 for LS, MK, LWA, HWA, RCA-1, and RCA-2 were 58, 53, 21, 55, 56, and 26 MPa, respectively. While the coarse aggregates used in Mix 1 (LS, MK, HWA, RCA-1) had different physical and mechanical characteristics, the compressive strength was reasonably comparable (53–58 MPa). This implied that the failure mechanism was dictated by the failure of the matrix and ITZ, as the failure plane initiated at the weakest element of the ITZ and caused a separation between the matrix and the aggregates, which was initiated at the level of the ITZ. The cracking was then propagated as the axial stress increased and imposed transverse stress around the aggregate particle causing the eventual failure of the concrete specimen. 

The superior performance of RCA-1 compared to RCA-2 was owing to the quality of mortar attached on the surface of coarse aggregate particles, which was characterized by the lesser volume of porous attached mortar, lesser water demand or absorption, and better abrasion resistance. Nevertheless, the existence of a high volume of porous mortar attached to the surface RCA-2 particles introduced an additional phase to concrete, and the failure mechanics were controlled by the bonding strength of the new ITZ to the old ITZ adhered. This zone was considered the weakest link, owing to the high volume of the porous adhered mortar and high absorption level of 8.5%. Consequently, microcracks initiated and propagated around that zone caused a separation between the matrix and RCA particles. 

The lowest compressive strength was recorded for LWA (21 MPa). It should be noted that the LWA aggregate was very porous, had substantially higher water absorption of 10.6%, and substantially lower abrasion resistance with a mass loss of 45%. The compressive strength drop of the LWA was attributed to a very low coarse aggregate strength compared to the mortar. As the LWA was subjected to the axial compressive loads, stress redistribution within the concrete specimen occurred, and due to the lower stiffness of the LWA, the matrix attracted more stress. The compressive strength of the LWA concrete specimen would be less than the strength achieved if this specimen contained only mortar. This phenomenon has been generally cited in the literature as the limit strength, which corresponds to concrete compressive strength assuming the elastic modulus of both the mortar and coarse aggregate is similar [3,4,7,9,10]. 

The role of coarse aggregate types and their contribution to the concrete compressive strength can be noticed for Mix 2, as presented in Figure 2. While the compressive strength enhanced for all concrete specimens when the amount of cement was increased from 300 to 500 kg/m^3^ and when the ratio of w/c ratio was reduced from 0.5 to 0.3, the degrees of compressive strength improvement for LS, MK, LWA, HWA, RCA-1, and RCA-2 were 20%, 28%, 103%, 48%, 10%, and 65%, respectively. The compressive strength values ranged from 41.7 MPa to 81.1 MPa. It was generally observed that a stiffer coarse aggregate produced a higher compressive strength. The LS and MK specimens, which comprised normal weight coarse aggregates, had an almost similar compressive strength of 70 and 68 MPa, respectively. While the MK aggregate had slightly higher abrasion resistance than that of the LS, the surface of the LS aggregate was rough and more angular than that of the MK, whose texture was somewhat smooth. The HWA achieved the highest compressive strength of 81 MPa, owing to its higher mechanical characteristics. The failure mechanism for the concrete produced using LS, MK, and HWA was controlled by the mechanical properties of coarse aggregate. The cracks go through the aggregates instead of moving through the ITZ due to the inherently stronger matrix in the case of Mix 2. As for the RCA-1, there was a slight improvement of 10% in compressive strength. This can be justified because of the adhered mortar, which introduced an additional phase to the composite phases. It is hypothesized that the adhered mortar still had an effect even though the recycled aggregate was obtained from HSC. Ideally, if RCA-1 had no adhered mortar, it would behave as LS since the HSC specimen from which RCA-1 aggregate was acquired was the same as LS aggregate. 

**Figure 3:**
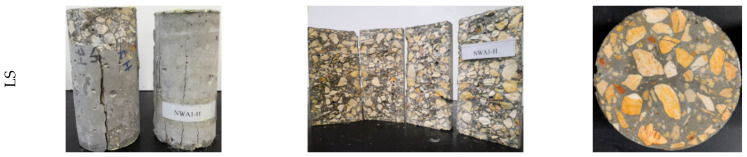

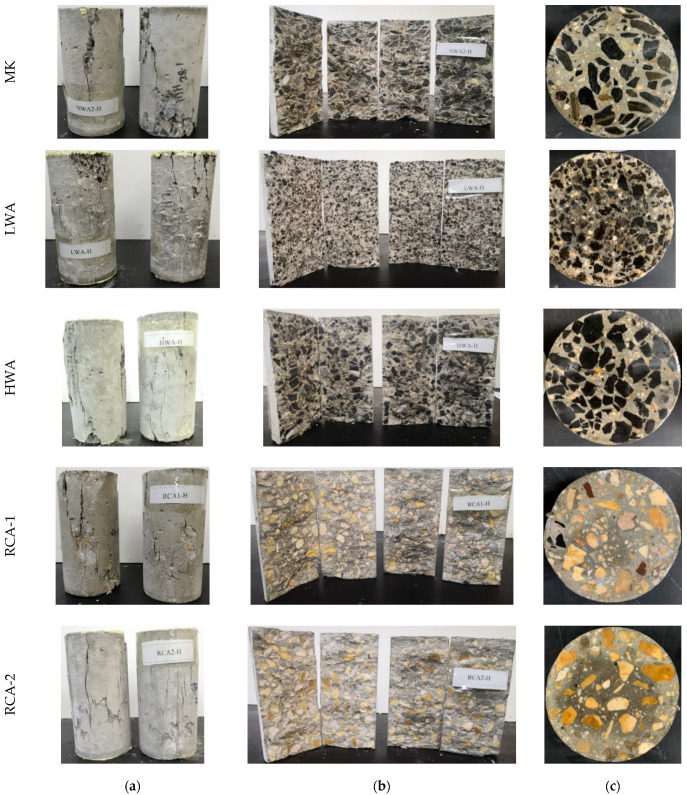
Illustrative photographs of Mix 1: (**a**,**b**) compression and splitting tension failure; and (**c**) cross section.

**Figure 4:**
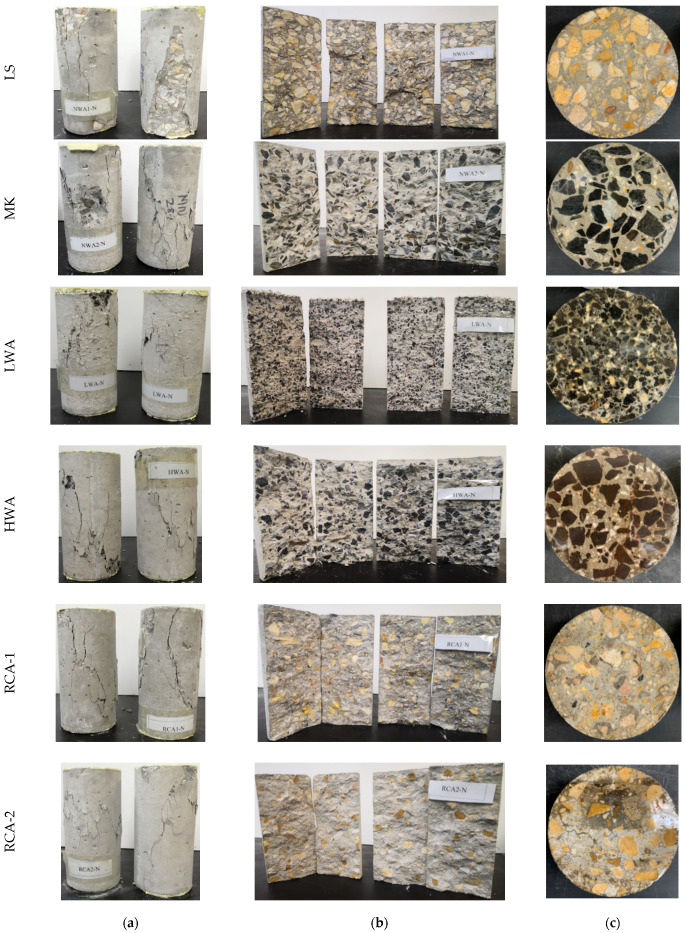
Illustrative photographs of Mix 2: (**a**,**b**) compression and splitting tension failure; and (**c**) cross section.

On the other hand, if the adhered mortar is very porous and has a higher absorption rate, it would typically act as RCA-2. Therefore, the characteristics of adhered mortar would generally dictate the performance of concrete. Even if the RCAs were procured from HSC, micro-cracking would initiate at the interface between the new ITZ and the old ITZ. The micro-cracks would then propagate until failure. The performance of RCA-2 was improved when the mix was rich (i.e., a lower ratio of w/c and higher cement quantity). However, this improvement was limited because of the low quality of adhered mortar. It should be noted that the coarse aggregate used in RCA-2 was obtained from low-quality concrete of a strength of about 20 MPa. The strength limitation was attributed to the properties of the RCA-2 aggregate, which had low abrasion resistance, high water absorption, and porous adhered mortar. While the compressive strength of the LWA concrete specimen was approximately doubled in Mix 2 compared to Mix 1 due to the richness of the concrete mixture, the LWA had the lowest compressive strength of 40 MPa. Unlike Mix 1, the bonding between the LWA and matrix was greatly improved, and hence the axial stresses were redistributed, and the interface could carry the higher compressive stress. As long as the tensile strength of LWA was reached, which typically occurred early for LWAs as opposed to NWAs, the failure occurred without substantial cracking in the matrix. At this stage, the concrete reached its ceiling strength, and no further gain in strength could be attained even with the rich mix (Mix 2). Figure 3 and Figure 4 exhibit the compressive failure pattern of the concrete cylinders with various types of coarse aggregates for both Mix 1 and Mix 2. In general, the failure mechanism previously described was very consistent with the observed failure pattern. 

### 3.3. Splitting Tensile Strength 

**Figure 5:**
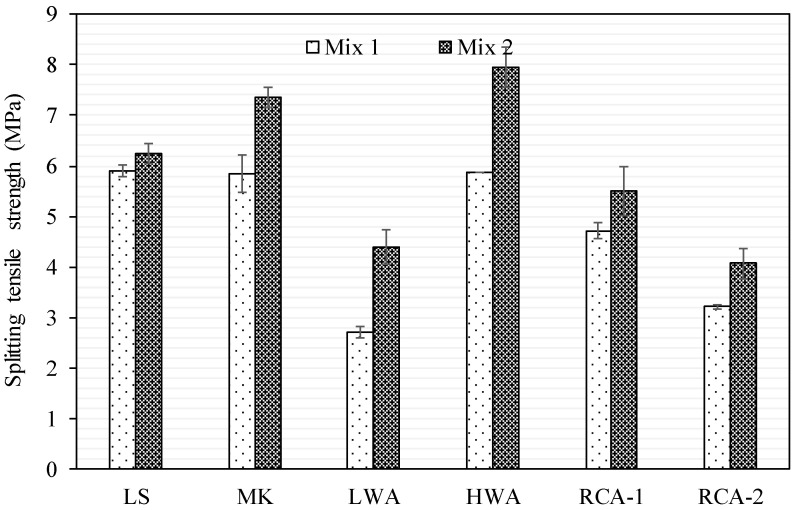
Splitting tensile strength results for Mix 1 and Mix 2.

The splitting tensile strength of concrete specimen tests was carried out in accordance with the ASTM C496/C496M-17 [34] for Mix 1 and Mix 2, as presented in Figure 5. Generally, the splitting tensile strength improved as the w/c ratio was reduced and as the cement amount was increased, and stiffer coarse aggregates seemed to produce higher tensile strength. The splitting tensile strengths for Mix 1 cast with LS, MK, LWA, HWA, RCA-1, and RCA-2 aggregates were 5.89, 5.83, 2.72, 5.86, 4.72, and 3.25 MPa, respectively. The tensile-to-compressive strength ratio of concrete specimens varied from 8% to 13%. Concerning Mix 2, the tensile strength was related to the coarse aggregate characteristics, as the matrix and ITZ were not the controlling parameters. Hence, stiffer aggregate appeared to have higher splitting tensile strength. The degrees of improvement in the splitting tensile strength for Mix 2 compared to Mix 1 were 6%, 26%, 62%, 36%, 17%, and 26% for the LS, MK, LWA, HWA, RCA-1, and RCA-2 aggregates, respectively. The ratio of splitting tensile strength to compressive strength was consistent, ranging from 9% to 11%. The effect of adhered mortar of RCA-1 and RCA-2 was estimated by correlating the splitting tensile strength with the LS, as the virgin coarse aggregate was the same. The existence of attached mortar for the RCA-1 and RCA-2 for Mix 1 resulted in degradation of the tensile strength by 20% and 45%, respectively (as compared to LS). However, the effect of the adhered mortar was less pronounced for Mix 2, with a reduction in the tensile strength of RCA-1 and RCA-2 by 12% and 35%, respectively. Figure 3 and Figure 4 exhibit the tensile failure pattern of the concrete cylinders with various types of coarse aggregates for both Mix 1 and Mix 2. In general, the failure mechanism previously described was consistent with the observed failure pattern.

### 3.4. Stress–Strain Relationship 

**Figure 6:**
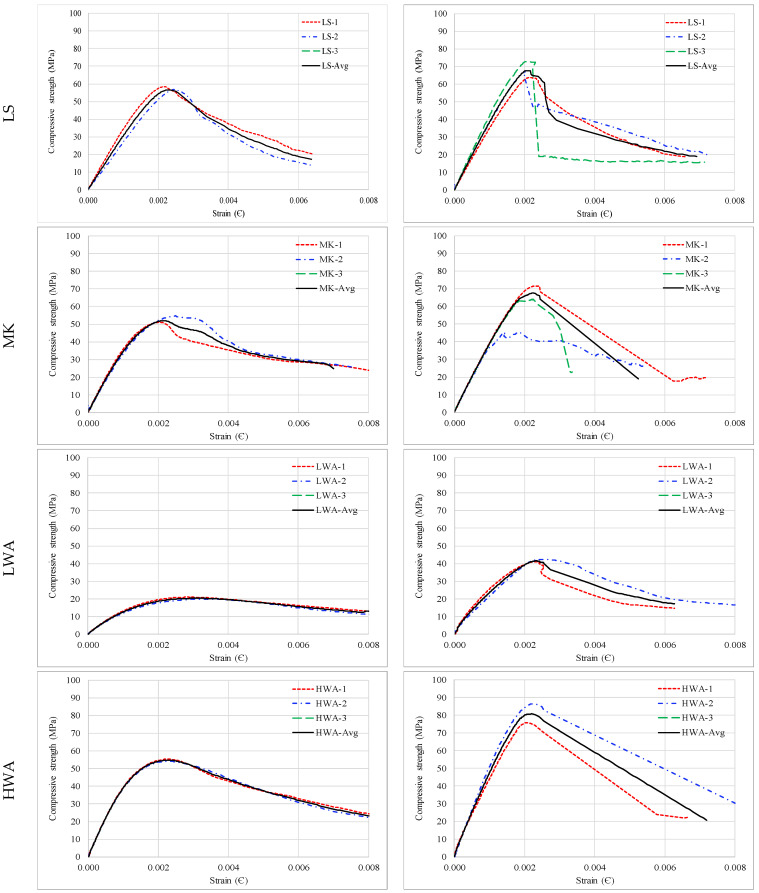

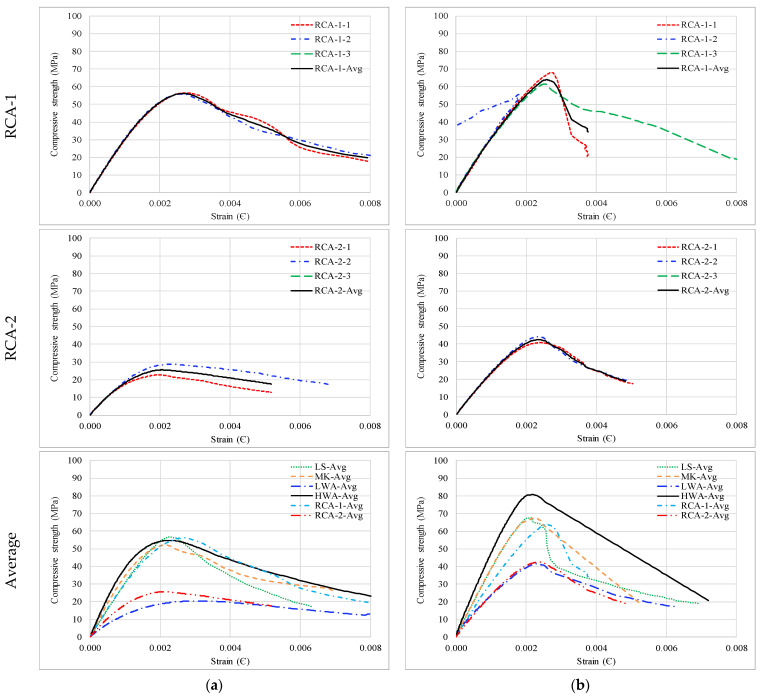
Stress–strain variation for: (**a**) Mix 1; and (**b**) Mix 2.

The uniaxial compressive stress–strain curves for Mix 1 and Mix 2 are displayed in Figure 6. A compressometer consisting of two rigid circular rings and two vertical linear variable differential transformers (LVDTs) on opposite sides attached to a data logger was used to measure the experimental strains up to the failure of specimens. The ascending branch of the stress–strain curves for all specimens appeared to be consistent with the specimens of the same mixture. However, the variations started to be noticeable in the post-peak region on the descending branch for the specimens whose compressive strength was greater than 60 MPa. The average determined from three cylinders for each concrete mixture helped to account for the variability between the specimens. Overall, the lower the compressive strength, the more ductile-type behavior is seen, and vice versa.

### 3.5. Strain at Peak Stress

**Figure 7:**
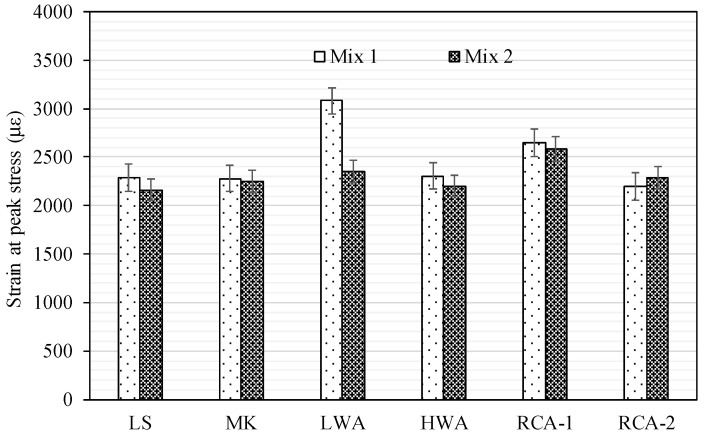
Strain at peak stress for Mix 1 and Mix 2.

The strain at peak stress is the main parameter of the stress–strain response of concrete. The strain at peak stress for Mix 1 and Mix 2 is shown in Figure 7. In general, strain at peak stress decreased as the compressive strength increased regardless of the coarse aggregate type. The strain at peak for Mix 1 and Mix 2 ranged from 2085 to 3080 microstrains and from 2160 to 2585 microstrains, respectively. The difference in the strain at peak stress between Mix 1 and Mix 2 for LS, MK, HWA, RCA-1, and RCA-2 was minor, with a difference of less than 5%. However, the LWA showed a high reduction of 25% in the strain at peak stress between Mix 1 and Mix 2. It is indicated that the strain at peak stress in the LWAs is typically higher than that of NWAs at the same level of compressive strength due to the lower modulus of elasticity of LWAs. The ratio of the strain at peak stress to the compressive strength for LS, MK, LWA, HWA, RCA-1, and RCA-2 mixes produced with rich mix (Mix 2) were 32.0, 33.2, 56.6, 27.2, 40.5, and 54 (microstrain/MPa), respectively. The increase in the strain of RCA-2 concrete compared to RCA-1 is due to the presence of porous mortar on the surface of recycled aggregates. It can be concluded that when the failure of concrete is dictated by the fracture of coarse aggregate, the strain at the peak is relatively comparable between the specimens, regardless of the coarse aggregate type. Nevertheless, there was no clear trend for the concrete specimens in which the ITZ and/or the matrix was the controlling factor.

### 3.6. Modulus of Elasticity 

**Figure 8:**
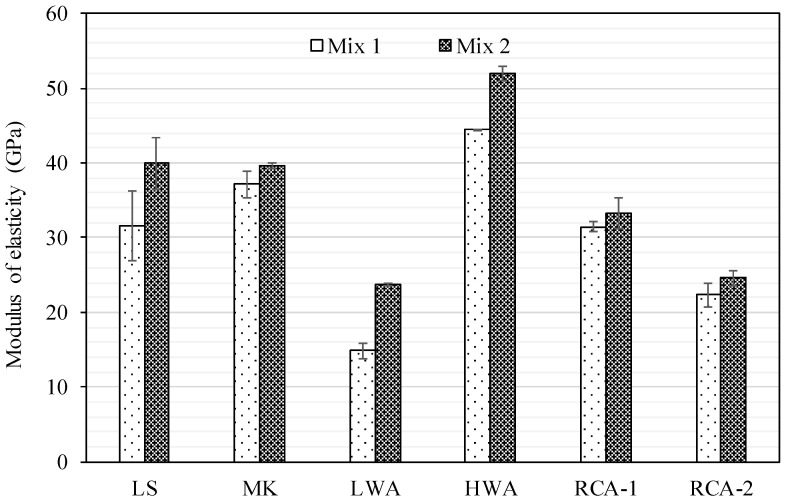
Modulus of elasticity results for Mix 1 and Mix 2.

The modulus of elasticity obtained in accordance with the ASTM C469 [35] for Mix 1 and Mix 2 concrete specimens is shown in Figure 8. In general, the modulus of elasticity is greater for Mix 2 than Mix 1 and is consistent with the trends observed in the compressive and splitting tensile strengths. While the modulus of elasticity is typically calculated based on the compressive strength, the coarse aggregate characteristics appear to have a significant contribution. As expected, it was generally noticed that the stiffer the coarse aggregate, the higher the elastic modulus. Although the compressive strength of LS, MK, HWA, and RCA-1 mixes were very comparable for Mix 1, the modulus of elasticity did not follow the same trend and was 31.6, 37.1, 44.5, and 31.5 GPa, respectively. The significant variations in the modulus of elasticity were attributed to the mechanical performance of coarse aggregate. When concrete is subjected to axial load, the stresses are distributed between the matrix and coarse aggregate based on their stiffness. Therefore, stiffer aggregate tends to resist higher stress leading to higher modulus. This trend was observed for the HWA, which achieved the highest modulus of elasticity (44.5 GPa) followed by MK (37.1 GPa), although they had very comparable compressive strength. However, the lowest modulus of elasticity was recorded for the LWA and was attributed to the lower concrete strength and the brittleness of coarse aggregate. While the RCA-1 behaved in a manner similar to the LS, the RCA-2 had the second-lowest modulus of elasticity, which was equivalent to 70% of that of the LS. This reduction was also attributed to the low quality of adhered mortar, which negatively influenced the compressive strength and consequently resulted in a lower modulus of elasticity. The improvements in the elastic modulus of Mix 2 compared to Mix 1 for the following aggregate types: LS, MK, LWA, HWA, RCA-1, and RCA-2 were 26%, 7%, 60%, 17%, 5%, and 10%, respectively. The increase in elastic modulus was due to the enhancement of the matrix strength, which along with the coarse aggregate, also contributed to the increase in the elastic modulus. It should be pointed out that the combined effect of reducing the ratio of w/c and increasing cement quantity (Mix 2) was more pronounced on the compressive strength than the elastic modulus.

The experimentally measured elastic modulus was compared with the predictive expressions available in the literature and adopted by various design codes to assess their validity for predicting the modulus of elasticity of concrete made using different coarse aggregate types. Table 4 lists and describes the formulas used as the basis of comparison with the experimental results obtained from the current study.

It can be seen from Table 5 and Table 6 that CEB-FIP [40] and BS EN-92 [41] did not yield a good prediction of the elastic modulus. The discrepancies in these models with the experimental values were significantly noticeable in the LWA and HWA since the elastic modulus predicted by CEB-FIP [40] was applicable only for concrete with a density of 2500 kg/m^3^. Moreover, there were no correction factors to account for the unit weight of lightweight concrete or the toughness of LWA. For instance, the elastic modulus of the LWA concrete of Mix 1 and Mix 2 predicted by the CEB-FIP-90 model [40] compared to the experimental value was overestimated by up to 84% and 55%, respectively. However, incorporating the correction factor for lightweight concrete in the BS-EN [41] formula reduced the overestimation of the predicted modulus of elasticity of LWA concrete for Mix 1 and Mix 2 compared to the CEB-FIP-90 equation to 55% and 28%, respectively. The modulus of elasticity computed by the BS 8110-2 [43] equation generally underestimated the elastic modulus by up to 30%, which was attributed to the conservative factor for the recommended modulus of elasticity of the aggregate (*K_o_*, which was taken as 20 kN/m^3^ for normal weight concrete). It should be noted that a range of *K_o_* values is provided, but the appropriate selection of a *K_o_* value is entirely left to the experience of designers. The ACI-318-19 [37] overestimated the modulus of elasticity of RCA-2 concrete by 25%, whereas the modulus of elasticity of MK concrete was underestimated by around 8%. 

The density-based formula of ACI-318-19 [37] generally overestimated the elastic modulus by 5% to 29%. This overestimation was attributed to not taking into consideration the contribution of the modulus of elasticity of aggregate into its formula. In its commentary, the design code ACI-318-19 [37] indicated higher variation in the calculated modulus of elasticity compared with that of the experimentally measured for compressive strength of 55 MPa or higher. While the existing stress–strain models in the literature were developed for normal weight concrete, Yang et al. [44] presented a stress–strain model considering a number of various compressive strengths and densities. A lower modulus of elasticity was observed for lightweight concrete [45], whereas an increase in the modulus of elasticity was reported for concrete of higher density [46]. Noguchi et al. [47] suggested that the expression of the modulus of elasticity can be represented in terms of the strength grade of concrete, density, mechanical properties of aggregate, and the use of supplementary cementing materials.

While the modulus of elasticity computed by AS-3600-09 [42] appeared identical to that of the density-based formula of ACI-318-19 [37] for compressive strength of less than 40 MPa, a slight modification was introduced to account for HSC. AS-3600-09 [42] normally resulted in a better prediction of the elastic modulus than that of the density-based formula of ACI-318-19 [37], and the differences between the calculated and measured elastic modulus did not exceed 15%, except for LWA from Mix 1 and RCA-2 from Mix 2. The ACI 363R-92 and CSA A23.3-04 equations showed great similarity in their coefficients, and thus the modulus of elasticity predicted by these code equations closely matched the experimentally measured results. The variations between the predicted and measured modulus of elasticity in ACI 363R-92 [38] and CSA A23.3-04 [39] were limited to 9% except for LWA concrete of Mix 1 (19%) and RCA-2 concrete of Mix 2 (29%). While the original source from which the RCA-1 and RCA-2 were obtained was the same, their modulus of elasticity results were significantly different due to the quality of the adhered mortar. Although the incorporation of the concrete density of coarse aggregates into design code equations improves the prediction of the modulus of elasticity, additional factors should be included to account for the strength, quality, and mineralogy of coarse aggregates. Findings imply a significant contribution of the coarse aggregate density towards calculating the modulus of elasticity.

The quality of the RCA is typically expressed in terms of water absorption, abrasion resistance, adhered mortar, and quality of original concrete from which RCA was obtained. Therefore, a valid model for concrete produced using RCA should consider these parameters in future studies.

### 3.7. Water Absorption

**Figure 9:**
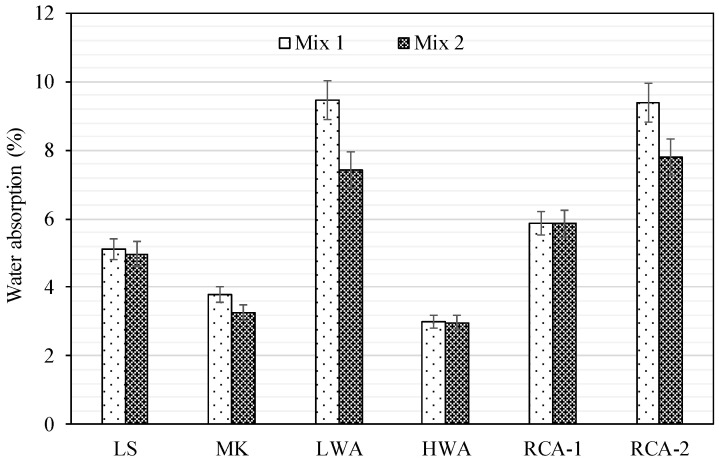
Water absorption of concrete for Mix 1 and Mix 2.

Water absorption, which represents the maximum value of water ingress, is considered an indirect measurement of water-accessible porosity and has been used to give some indication of the permeability and durability of concrete. The water absorption test of concrete specimens was carried out as per the ASTM C1585-20 [36] for Mix 1 and Mix 2; the results are presented in Figure 9. The water absorption test determines the difference in mass between a dry state and a saturated surface dry (SSD) state following the immersion in water for a given amount of time. Although the water absorption of concrete is primarily dependent on the ratio of water to binder (w/b) [48], the coarse aggregate characteristics appeared to play a major role in concrete water absorption. The results indicated that there was a small effect of less than 3% on concrete water absorption for the LS, MK, HW, and RCA-1 between Mix 1 and Mix 2. This was attributed to the lower water absorption of the coarse aggregates in these concrete mixtures. However, the reduction in water absorption of LWA and RCA-2 in Mix 2 was more pronounced than in Mix 1, with a reduction in concrete water absorption of 28% and 20%, respectively. The effect of attached mortar for RCA-1 and RCA-2 compared to the LS, which all had the same virgin source, on the water absorption was clearly more noticeable in RCA-2 than that of RCA-1 with a rise in water absorption by 15% and 148%, respectively, for Mix 1 and by 17% and 138%, respectively for Mix 2. The findings indicate the poor quality of the attached mortar of RCA-2, which had a high volume of porous mortar attached on the surface of RCA-2 particles causing higher water absorption compared to RCA-1. This seemed to be very consistent with the physical properties of RCA-2, with a water absorption of 9.33%. 

The study is expected to serve as a benchmark for comparing the performance of different types of coarse aggregates covering a broad range of mineralogy and densities. The test results indicated that HSC (compressive strength > 40 MPa) could be produced using rich mix even with the LWA and poor-quality recycled concrete-based normal weight aggregate RCA-2. Additionally, water absorption can also be considerably reduced for these highly porous aggregates. The test results are also expected to serve as a guide for optimizing the mixes using different available aggregates.

## 4. Conclusions 

The performance of concrete produced using various recycled construction and by-product industrial wastes, manufactured limestone, quartzite, and volcanic scoria with densities varying from 860 to 2300 kg/m^3^ was investigated. Compressive strength, splitting tensile strength, stress–strain relationships, and water absorption were used to assess concrete performance. To determine the coarse aggregate contribution to the overall concrete performance, lean and rich concrete mixtures (Mix 1 and Mix 2) were used. Mix 1 had a water-to-cement ratio (w/c) of 0.5 and a cement quantity of 300 kg/m^3^, whereas a higher quantity of cement of 500 kg/m^3^ and a lower ratio of w/c of 0.3 were used for Mix 2. Based on the outcomes of the experimental study results, the following conclusions can be drawn:

1. The contribution of coarse aggregate towards the concrete strength is more pronounced for Mix 2 as the coarse aggregate characteristics controlled the failure mechanics. While the LS, MK, HWA, and RCA-1 had comparable compressive and tensile strengths for Mix 1, the elastic modulus was greatly influenced by the abrasion resistance and strength of coarse aggregates.
2. The combined effect of reducing the ratio of w/c and increasing the quantity of cement improved (Mix 2 vs. Mix 1) the mechanical performance of concrete irrespective of the coarse aggregate type. The rate of increase in compressive strength for Mix 2 relative to Mix 1 was more pronounced than splitting tensile strength and the modulus of elasticity.
3. The density of coarse aggregate was found to have an influence on the concrete mechanical properties. It should also be noted that the stiffer the aggregate, the more likely to enhance concrete performance.
4. The mechanical characteristics of concrete with recycled aggregates were greatly dependent on the quality of attached mortar on the surface of coarse aggregate. However, the existence of attached mortar may have some impact on its mechanical properties.
5. The LWA resulted in lower mechanical performance than LS, MK, HWA, and RCA-1 due to the higher volume of porosity, higher mass loss, and weaker mechanical strength of coarse aggregate. While the rich mix (Mix 2) improved the matrix and ITZ characteristics, the strength of the concrete reached a ceiling limit beyond which no further increase would be expected.
6. The low quality of adhered mortar significantly resulted in a drop in the concrete performance. The drop in RCA-2 compressive strength compared to RCA-1 was 49% for Mix 1 and 31% for Mix 2.
7. The experimentally measured modulus of elasticity was compared with available formulas of design codes. ACI 363 R-92 and CSA A23.3-04 were the best prediction models, followed by the density-based formulas of ACI-318-19 and AS-3600-09.
8. Irrespective of w/c ratios, the water absorption of concrete was high for concrete whose coarse aggregates had high water absorption.
9. While the density of concrete is considered an essential factor in predicting the modulus of elasticity, the mechanical characteristics of coarse aggregate also appear to have a great influence. Although a majority of design codes impose an upper limit on the density of concrete (2500 kg/m^3^), the modulus of elasticity of HWA concrete correlates well with the design equations of ACI 363R-92 and CSA A23.3-04. Thus, the modulus of elasticity of HWA concrete can be appropriately computed using the equations of these codes.
10. Future research is needed to evaluate the effect of steel and synthetic fibers on the performance of concrete with recycled construction and by-product industrial waste coarse aggregates.

## Figures and Tables

**Table 1:** Fine and coarse aggregates physical and abrasion properties.

Aggregate Type	Specific Gravity	Water Absorption (%)	Density of Aggregate (kg/m^3^)	Los Angeles Abrasion (%)
Fine aggregate (FA)	2.60	1.30	1645	-
LS	2.61	1.20	1575	23.1
MK	2.72	1.25	1487	19.8
LWA	1.72	10.60	860	45.0
HWA	3.70	1.17	2300	13.3
RCA-1	2.44	4.77	1610	28.4
RCA-2	2.32	9.33	1555	37.5

**Table 2:** Concrete mixture design of Mix 1.

Concrete Constituents	LS	MK	LWA	HWA	RCA-1	RCA-2
w/c ratio	0.5	0.5	0.5	0.5	0.5	0.5
Cement (kg/m^3^)	300	300	300	300	300	300
Water (kg/m^3^)	150	150	150	150	150	150
Coarse aggregate (kg/m^3^)	1167	1232	692	1649	1041	958
Fine aggregate (kg/m^3^)	792	792	792	792	792	792
Unit weight of concrete, *w_c_* (kg/m^3^)	2443	2498	2025	2918	2349	2295

**Table 3:** Concrete mixture design of Mix 2.

Materials	LS	MK	LWA	HWA	RCA-1	RCA-2
w/c ratio	0.3	0.3	0.3	0.3	0.3	0.3
Cement (kg/m^3^)	500	500	500	500	500	500
Water (kg/m^3^)	150	150	150	150	150	150
Coarse aggregate (kg/m^3^)	1082	1129	634	1509	955	875
Fine aggregate (kg/m^3^)	724	724	724	724	724	724
Unit weight of concrete, *w_c_* (kg/m^3^)	2467	2526	2092	2909	2390	2340

**Table 4:** Modulus of elasticity prediction models in various building codes.

Design Code	Code Equation for Predicting Modulus of Elasticity of Concrete, *E_c_*	Limitations and Coefficients
ACI-318-19 [37]	$E_{c}=4700\sqrt{f_{c}^{'}}$	For normal weight concrete
ACI-318-19 [37] (density-based formula)	$E_{c}=0.043w_{c}{}^{1.5}\sqrt{f_{c}^{'}}$	$1440<w_{c}<2560$ kg/m^3^ Higher discrepancies between measured and calculated $E_{c}$ when $f_{c}^{\text{'}}\geq 55$ MPa
ACI 363R-92 [38]	$E_{c}={\left(\frac{w_{c}}{2320}\right)}^{1.5}\left(3320\sqrt{f_{c}^{'}}+6890\right)$	$21<f_{c}^{'}<83$ MPa $1500<w_{c}<2500$ kg/m^3^
CSA A23.3-04 [39]	$E_{c}={\left(\frac{w_{c}}{2320}\right)}^{1.5}\left[3300\sqrt{f_{c}^{'}}+6900\right]$	$1500<w_{c}<2500$ kg/m^3^
CEB-FIP-1990 [40]	$E_{c}=E_{co}\;\alpha {\left(\frac{f_{c}^{'}}{f_{cmo}}\right)}^{\frac{1}{3}}$	$f_{c}^{'}\leq 50$ MPa $E_{co}=21500$ MPa $f_{cmo}=10$ MPa $w_{c}=2500$ kg/m^3^ α = 0.7 for sandstone aggregates, 0.9 for limestone aggregates, 1.0 for quartzite aggregates, and 1.2 for dense limestone aggregates and basalt.
BS EN 1992 [41]	$E_{c}=22000k{\left(\frac{w_{c}}{2200}\right)}^{2}{\left(\frac{f_{c}^{'}}{10}\right)}^{0.3}$	k = 1.2 for basalt aggregates, 1.0 for quartzite aggregates, 0.9 for limestone aggregates, 0.7 for sandstone aggregates. for LWA, multiply by ${\left(\frac{w_{c}}{2200}\right)}^{2}$
AS-3600-2009 [42]	$E_{c}=0.043w_{c}{}^{1.5}\sqrt{f_{c}^{'}}$ for $f_{c}^{'}<40$ MPa $E_{c}=w_{c}{}^{1.5}\left(0.024\sqrt{f_{c}^{'}}+0.12\right)$ for $f_{c}^{'}>40$ MPa	$1500<w_{c}<2500$ kg/m^3^
BS 8110-2 [43]	$E_{c}={\left(\frac{w_{c}}{2400}\right)}^{2}\left(K_{o}+0.2f_{c}^{'}\right)$	$K_{o}=14\;\mathrm{to}\;26$ kN/mm^2^ For LWA, multiply by ${\left(\frac{w_{c}}{2400}\right)}^{2}$

*w_c_* is the density of concrete in kg/m^3^, $f_{c}^{'}$ is the compressive strength of concrete in MPa.

**Table 5:** Experimentally measured modulus of elasticity and calculated modulus of elasticity by various design codes.

Mix	Aggregate Types	Experimental Results (GPa)	ACI [37] (GPa)	ACI density-Based Formula [37] (GPa)	ACI 363 [38] (GPa)	CEB-FIP [40] (GPa)	BS EN 1992 [43] (GPa)	CSA A23.3 [39] (GPa)	AS-3600 [42] (GPa)	BS 8110-2 [41] (GPa)
Mix 1	LS	31.62	35.82	39.35	34.60	34.78	33.56	34.44	36.37	31.62
	MK	37.10	34.20	39.06	34.70	37.47	36.27	34.53	36.78	30.59
	LWA	14.87	-	17.73	17.88	27.30	23.11	17.80	17.73	17.15
	HWA	44.46	-	50.14	44.38	45.47	43.96	44.17	46.90	30.94
	RCA-1	31.47	35.25	36.74	32.42	34.41	33.24	32.26	34.17	31.25
	RCA-2	22.34	23.86	24.02	23.39	26.53	26.30	23.29	24.02	25.15
Mix 2	LS	39.96	39.29	44.04	38.00	36.99	35.48	37.81	39.29	33.98
	MK	39.57	38.70	44.95	38.89	40.69	39.06	38.71	40.32	33.56
	LWA	23.75	-	26.55	24.26	34.59	30.52	24.15	26.30	21.53
	HWA	51.99	-	61.06	51.92	51.84	49.47	51.66	52.99	36.23
	RCA-1	33.20	36.89	39.44	34.46	35.48	34.17	34.30	36.03	32.32
	RCA-2	24.58	30.63	31.72	28.91	31.34	30.56	28.78	31.29	28.50
Maximum		51.99	39.29	61.06	51.92	51.84	49.47	51.66	52.99	36.23
Minimum		14.87	23.86	17.73	17.88	26.53	23.11	17.80	17.73	17.15

**Table 6:** Comparisons between the experimentally measured and predicted modulus of elasticity by various design codes.

Mix	Aggregate Types	ACI/Experimental Results	ACI Density-Based Formula/Experimental Results	ACI 363/Experimental Results	CEB-FIP/Experimental Results	BS EN 1992/Experimental Results	CSA A23.3/Experimental Results	AS-3600/Experimental Results	BS 8110-2/Experimental Results
Mix 1	LS	1.13	1.24	1.09	1.10	1.06	1.09	1.15	1.00
	MK	0.92	1.05	0.94	1.01	0.98	0.93	0.99	0.82
	LWA	-	1.19	1.20	1.84	1.55	1.20	1.19	1.15
	HWA	-	1.13	1.00	1.02	0.99	0.99	1.05	0.70
	RCA-1	1.12	1.17	1.03	1.09	1.06	1.03	1.09	0.99
	RCA-2	1.07	1.07	1.05	1.19	1.18	1.04	1.07	1.13
Mix 2	LS	0.98	1.10	0.95	0.93	0.89	0.95	0.98	0.85
	MK	0.98	1.14	0.98	1.03	0.99	0.98	1.02	0.85
	LWA	-	1.12	1.02	1.46	1.28	1.02	1.11	0.91
	HWA	-	1.17	1.00	1.00	0.95	0.99	1.02	0.70
	RCA-1	1.11	1.19	1.04	1.07	1.03	1.03	1.09	0.97
	RCA-2	1.25	1.29	1.18	1.28	1.24	1.17	1.27	1.16
Maximum		1.25	1.29	1.20	1.84	1.55	1.20	1.27	1.16
Minimum		0.92	1.05	0.94	0.93	0.89	0.93	0.98	0.70

## Data Availability

Not applicable.
